# Analysis of Risk Factors and Assessment of miRNA-122 as a Novel Biomarker for Colon Cancer in Egyptian Patients

**DOI:** 10.31557/APJCP.2026.27.1.337

**Published:** 2026-01-22

**Authors:** Mohamed A.A Bassiony, Mohamed G Hamed, Marwa M Esawy, Ayman FE Mohamed

**Affiliations:** 1 *Internal Medicine Department, Faculty of Medicine, Zagazig University, Zagazig, Egypt.*; 2 *Internal Medicine Department, Vision Colleges in Riyadh, Riyadh, Saudi Arabi. *; 3 *Clinical Pathology Department, Faculty of Medicine, Zagazig University, Zagazig, Egypt.*

**Keywords:** Colon cancer, NSAIDs, smoking, obesity, diabetes, dyslipidemia, miRNA-122

## Abstract

**Background:**

Colon cancer is one of the most prevalent cancers in the world; some of its risk factors are controllable. A healthy lifestyle and early diagnosis significantly reduce colon cancer incidence and mortality. The aim of this study was to assess the most important risk factors for colon cancer in the Egyptian patients as well as the usefulness of miRNA-122 as diagnostic biomarker for colon cancer.

**Methods:**

In this case control study, 102 colon cancer cases and equal number of controls were enrolled between April 2022 till June 2022 in two Egyptian centers. Both groups were assessed by a structured questionnaire, anthropometric measurements and serum levels of miRNA-122.

**Results:**

Dietary factors, older age, obesity, rural residence, prolonged use of non-steroidal anti-inflammatory drugs, and cigarette smoking were significantly associated with colon cancer in our study subjects. miRNA-122 demonstrated significant sensitivity and specificity for colon cancer diagnosis, with a cutoff value of 0.946.

**Conclusion:**

The most important risk factor for colon cancer in the Egyptian patients were smoking, obesity, diabetes, dyslipidemia and excessive processed meat intake with low vegetables consuption. Our study suggests miRNA-122 as a potential non-invasive diagnostic biomarker for colon cancer.

## Introduction

Colon cancer is the third most common cancer in men and women and the second most common cause of cancer-related mortality worldwide in both developed and developing countries, representing more than 9% of all cancer-related deaths [1-3]. In Egypt, colon cancer is the seventh most common cancer accounting for about 6.5% of all malignant tumors (3.5% of cancer in men and 3% of cancer in women), and 14% of colonoscopies detect the presence of colorectal cancer [4, 5].

The exact aetiology of colon cancer is still unknown. However, multiple risk factors have been identified in literature, particularly in the western communities. Some of these risk factors are non-modifiable, such as old age and hereditary polyposis and other risk factors are modifiable such as low physical activity, diet rich in fat and red meat, obesity, smoking, excess alcohol intake and inflammatory bowel disease. Lifestyle modifications may prevent up to 50% of cases of malignant tumors [6-9].

Cellular proliferation and differentiation are controlled by specific genes that in turn are under control of certain regulatory factors (tumor suppressors and oncogenes) which can switch these genes on and off. Most malignant tumors are initiated by imbalance between oncogenes and tumor suppressors causing mutations in genes with subsequent uncontrolled cellular proliferation and differentiation. MicroRNA (miRNA), a small, single stranded, noncoding RNA (ncRNA), composed of 1825 nucleotides, functions to suppress gene expression by stimulating the degradation or inhibiting the translation of messenger RNA (mRNA). They may act as biomarkers for the diagnosis and prognosis of multiple malignant tumors. miRNA122 is a key biomarker of the miRNAs. It is mainly expressed in liver tissues and involved in lipid and carbohydrate metabolism as well as development and progression of hepatocellular carcinoma. *miRNA-122* is also expressed in the tissue of colon cancer as it controls the expression of ALDOA (Aldolase, fructose-Bisphosphate A) gene, that is involved in the invasiveness of colon cancer and development of liver metastases [10-12].

In this study, the authors will investigate the validity of serum *miRNA-122* as a novel diagnostic biomarker for colon cancer as well as the most important risk factors of colon cancer in the Egyptian patients.

## Materials and Methods

Study design and participants: This case control study was conducted in accordance with the principles of the Declaration of Helsinki, and all participants provided written informed consent prior to enrollment. The study protocol was approved by the relevant ethical committees and institutional review board. It was conducted on 102 cases with colon cancer (diagnosed by colon biopsy after colonoscopy) recruited from oncology and gastroenterology units in two university hospitals, in the period between April 2022 and June 2022. In addition, an equal number of normal and healthy control subjects were randomly selected from the same hospitals during the same period. 67 control subjects were attending the hospital for routine pre-employment investigations, 26 control subjects for pre-university enrolment investigations, and 9 control subjects for routine pre-operative investigations. Control subjects who attended for pre-operative investigations included 6 patients for laparoscopic cholecystectomy and 3 subjects underwent surgical repair of hernia.

### Inclusion criteria

#### age

18-75 years, both males & females, confirmed colon cancer by pathology & radiology, and patients who agreed to be enrolled in the study 

### Exclusion criteria

History of previous chemotherapy, significant heart, liver, or kidney impairment, or the presence of other malignant tumors 

### Sample size calculation

The minimum sample size (n=94) was estimated according to:

$\mathrm{Sample size}=\frac{Z_{1-\frac{\alpha }{2}}^{2}P(1-p)}{d^{2}}$

Where: Z_1-α/2_= is the standard normal variate at 5% type 1 error (P<0.05) it is 1.96, P= the expected proportion based on previous studies (6.5%) and d= the absolute error (0.05) [4].

### Data collection

#### 1. Structured questionnaire

was used for data collection from the studied groups. Data were collected from patients’ medical files. Face-to-face interviews were performed with cases and control subjects to complete the missing data. Personal data (age, sex, residence, marital status, educational level, employment, and income), dietary habits (frequency of intake of red meat, processed meat, fatty meals, drinking milk, and intake of vegetables, fruits, cheese and eggs) and lifestyle habits (frequency of physical activity, smoking, constipation, use of non-steroidal anti-inflammatory drugs (NSAIDs) and history of hypercholesterolemia) were all included in the questionnaire [13-17].

### 2. Anthropometric measurements

The body mass index (BMI) was estimated after height and weight were measured while standing without shoes or bulky clothes. 

### 3. Laboratory investigation

Complete blood count, liver function tests, coagulation profile, and kidney function tests were done for all study subjects. A blood sample (3 ml) was collected from subjects of both groups by qualified nurses through venipuncture under complete aseptic condition into EDTA-containing tubes, then tubes were frozen in (-80oC) till analysis. One milliliter of EDTA blood samples were subjected to total RNA extraction using GENEzol™ Reagent Gene aid (Taiwan). Reverse transcriptase was used to form complementary DNA (cDNA) using TOPscript™ DryMIX (dT18/dN6 plus), enzynomics (South Korea), Synthesis Kit Reverse Transcription Kits. This was done using Applied Biosystems (Veriti 96 well thermal cycler), manufacturer: life technologies – Singapore, serial number: 2990226743. Amplification and detection of MiR-122 were performed by Applied Biosystems (step one Real Time PCR) serial number 271003648 using SYBR Green with high Rox enzynomics, TOPrealTM qPCR 2X PreMIX (SYBR Green with strong ROX) (South Korea). *miRNA-122* was extracted using PAXgen blood miRNA kit following the manufacturer’s instruction manual [18].

The gene sequence primers used were Forward Sequence GGAGTGTGACAATGGTG, Reverse Sequence GAACATGTCTGCGTATCTC

### Statistical analysis

It was done using version 16.0 of the Statistical Package for Social Science (SPSS Inc, Chicago, IL). The collected data were tabulated, summarized as categorical data, frequency and percentage then analyzed. The Chi-square test, Fishers exact test, T test, and Mann-Whitney U test were used as significant tests for comparing the two research groups. A logistic regression analysis was undertaken to identify the significant risk factors for colon cancer in our cases. A ROC curve was generated. A P-value less than or equal to 0.05 was deemed statistically significant.

## Results

Cases of colon cancer in this study were significantly older than controls (51.47±14.55 years vs 39.61±15.11 years; P<0.001). Male predominance was noticed among cases compared with controls (84.3% vs 62.7%; P<0.001). Incidence of residence in rural areas was considerably higher among cases compared with controls (49.0% vs 31.4%; P<0.001). Also, incidence of being married was considerably higher among cases compared with controls (98.0% vs 76.5%; P<0.001). Moreover, Cases had lower educational levels, higher unemployment rates, and lower income (all P<0.001). Colon cancer cases were found to have significantly shorter physical activity time/day (median time 2hrs/day vs 4 hrs/ day; P=0.01) and higher incidence of prolonged sitting time (66.7% vs 51%; P=0.23) when compared with controls (Table 1).

Our study cases were found to have higher incidences of constipation, hypercholesterolemia, more prolonged use of NSAIDs, type 2 DM, and cigarette smoking (All P values < 0.05) when compared with controls. No statistically significant difference was found between both groups regarding the family history of colon cancer. Regarding anthropometric measurements, colon cancer cases were found to be significantly shorter (median height 163cm vs 165; P=0.001) and have considerably higher BMI (median BMI 29.6 vs 28; P=0.022) when compared with controls (Table 2). The dietary risk factors for colon cancer were intake of red and processed red meats (37.3% and 76.5% for cases and 9.8% and 9.8% for control), frequent intake of milk (66.7%), cheese (54.9%), eggs (74.5%) and lower intake vegetables, and fruits (56.9%) (Table 3). 

**Figure 1:**
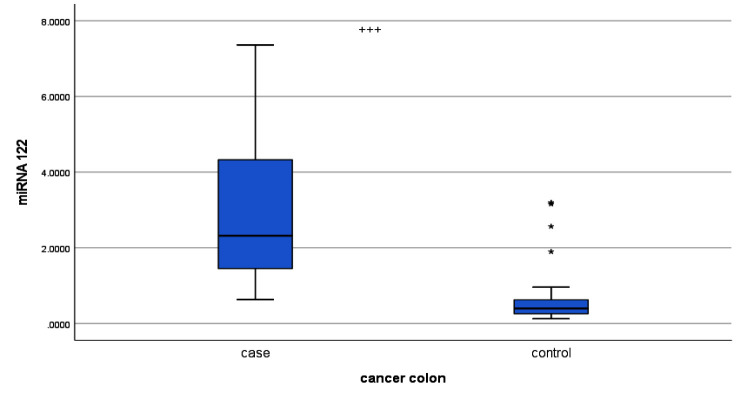
Box Plot of Comparison between Cases and Control Regarding *miRNA-122* Gene

**Figure 2:**
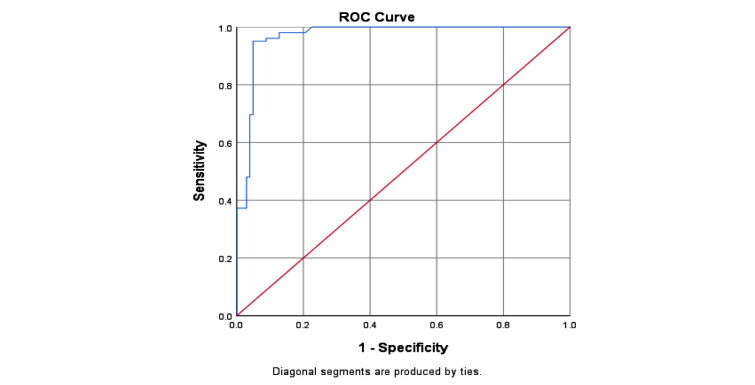
Roc curve for *miRNA-122* as a Diagnostic Biomarker in Colon Cancer

Expression level of *miRNA-122* was significantly higher among cases when compared with controls (2.3 vs.0.39; P<0.001) (Table 4) and (Figure 1). ROC curve analysis for *miRNA-122* revealed that the cut off value of 0.946, significantly predicts colon cancer with AUC: 0.968, sensitivity 95.0% and specificity 91.0%. The 95% confidence interval (95% CI) was 0.944-0.993. (Table 5) and (Figure 2).

Logistic regression analysis showed that higher BMI (OR=1.07, 95% CI=1.01- 1.015), low intake of vegetables and fruits (OR=3.7, 95% CI=1.35-10.1), older age (OR=1.13, 95% CI=1.07- 1.19), excess processed meat intake (OR=2.8, 95% CI=1.21- 6.53) and living in rural areas (OR=2.5, 95% CI=1.03-6.1) were the most important risk factor of colon cancer in our subjects, (P ≤ 0.05), (Table 6).

**Figure 3:**
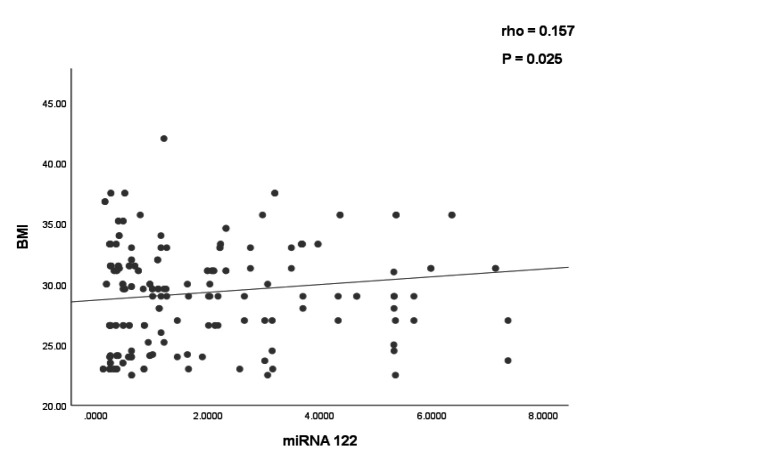
Scatter Diagram for the Relationship between Body Mass Index and *miRNA-122*

Scatter graph analysis showed a significant positive correlation between the higher BMI and *miRNA-122* (rho = 0.157 and P=0.025). (Figure 3)

## Discussion

Colon cancer is a significant cause of cancer-related morbidity and mortality worldwide. It has an increasing incidence in Egypt among both men and women as confirmed by colon biopsies during colonoscopy in gastroenterology units [5].

In our current study, the most important risk factors of colon cancer in the Egyptian patients and the validity of *miRNA-122* as a diagnostic biomarker for colon cancer were investigated. Our study recruited 102 cases of colon cancer from the oncology units in 2 Egyptian university hospitals. This group of cases was compared with an equal number of age and gender matched normal healthy controls who were randomly selected from the same hospital during the same period. 

In a retrospective study conducted by Ghodssi-Ghassemabadi et al., on newly diagnosed colon cancer patients from Iran, patients at diagnosis had a mean age of 52.87 ± 11.93 years. This result is similar to our study. This was also supported bythe findings of Cancer.Net Editorial Board where the majority of colon cancer cases occur in people older than 50 years old [19, 20].

In the current research, colon cancer was predominant in men. This finding is similar as stated in the United Kingdom (UK) by White et al., as well as the findings from the data collected by the National Cancer Institute’s Surveillance, the Centers for Disease Control and Prevention and National Program of Cancer Registries in United States of America (USA). Male predominance of colon cancer was explained by higher exposure to cigarette smoking among men, and the finding that elevated progesterone and estrogen levels were associated with lower risk of developing colon cancer in women as reported by American Cancer Society [21-24].

Colon cancer was substantially more common in married patients than unmarried subjects in the present study. This finding is consistent with the data from the National Health Interview Survey Analyses of 2000, 2005, and 2008 and the US Surveillance, Epidemiology, and End Results (SEER) Program [25, 26].

In the current study, the majority of colon cancer cases (86%) had only primary education and about 60% of cases didn’t have enough money for daily needs. This was in agreement with the results of Moroccan multicenter prospective cohort study that reported that a significant number of colon cancer patients were illiterate and the majority of them had a low-intermediate socio-economic status [27].

The results of our study showed a higher incidence of colon cancer with excess intake of red and processed meat as well as a decreased incidence of colon cancer in subjects who had a regular consumption of milk, fresh vegetables, fruits and regular physical activity for at least 30 minutes/day. These findings are comparable to those reported by Aykan & Kuipers et al. This can be explained by Meat contains essential anticancer nutrients such as selenium, zinc, omega-3 fatty acids, vitamins B6, B12, D, and folic acid. So, meat should not be excluded from the human diet. However, high-temperature cooking of red meat might generate carcinogenic heterocyclic amines and polycyclic aromatic hydrocarbons. Nitrite and N-nitroso compounds may present certain processed meat products. Therefore, the recommended weekly intake of meat is 500 grams (cooked weight), and processed meat should be avoided whenever feasible [28-30].

Frequent eating of fruits and vegetables protects against cancer via biological processes depending on phytochemicals, which influence the growth of many malignancies, and antioxidants, which minimize cellular damage produced by cancer-causing reactive oxygen species. Lastly, the fiber component of fruits and vegetables increases stool volume and lowers stomach transit time, hence decreasing exposure to carcinogens [31]. 

Our findings revealed that higher BMI was associated with a higher incidence of colon cancer in our subjects compared with controls. These results are in agreement with those of the American cancer society surveillance and El-Taher et al. this can be explained by that obesity causes imbalance in gut microbiota and induces chronic inflammatory response in the tissues including the colon. Also, excess leptin production and insulin resistance may contribute to the development of colon cancer as stated in case-control, hospital-based study that was conducted in KSA [24, 32, 33, 34, 35].

Our patients with colon cancer showed a higher incidence of chronic constipation. This finding goes in agreement with that reported by Alsheridah & Akhtar and Guérin et al. [36] This association between chronic constipation and colon cancer was attributed to prolonged colon transit times, which increased the interaction between intestinal mucosa with carcinogenic compounds, such as bile acids, fecapentaenes, and ammonium acetate, in the intestinal lumen. [13, 29, 37]

Furthermore, in concordance to the present study, there was a considerably higher risk for colon cancer development in patients with type 2 diabetes mellitus, prolonged use of NSAIDs and smokers compared with control subjects. This could be explained by the shared risk factors for both colon cancer and type 2 diabetes mellitus. Also, our results revealed no statistically significant difference between colon cancer cases and control subjects regarding the family history of colon cancer. El-Taher et al. [32] found similar results, however, they reported that family history of colon cancer was higher among cases when compared with controls, yet the difference was statistically non-significant. Regarding our statistical finding that the use of NSAIDs was significantly more in the cases than the control subjects, we believe that was most probably secondary to the significantly higher number of smokers in our cases than control subjects. NSAIDs & aspirin might have a protective effect against GIT malignancy including colon cancer. However, long-term smoking increases colon cancer by 30% despite NSAIDs use due to increased unstable microsatellites in colon cancer patients. Additionally, our Egyptian subjects usually use non-selective NSAIDs, not aspirin or COX2-inhibitors which believed to have more protective value against colon cancer [24, 34].

Discovery of ncRNAs and their role in regulating proteins expression was a major breakthrough in the current century. miRNAs are evolving as a promising class of therapeutic targets for multiple diseases including malignant tumors. miRNAs can act as either tumor suppressors or oncogenes and may be involved in proliferation, differentiation, migration, invasion and metastasis of malignant tumors. In the present study, serum levels of *miRNA-122* were significantly higher among cases when compared with controls (2.3 vs.0.39; P<0.001). This is in agreement with Maierthaler et al., Baranwal et al., and Chi et al., who reported that increasing miRNA -122 levels were associated with the pathogenesis as well as poor prognosis and liver metastasis of colon cancer [37-39]. 

The ROC curve in our study revealed that *miRNA-122* with a cut off value 0.946 significantly predicts colon cancer. The AUC was 0.968 with 95% sensitivity and 91% specificity and (95% C.I: 0.944- 0.993). This indicates that *miRNA-122* is a sensitive and specific test for the diagnosis and prognosis of colon cancer. This result is consistent with the findings of Chen et al. [40] who reported an AUC for miR-122 of 0.808 (95% CI, 0.712-0.905; P<0.01) in patients with metastatic gastric cancer. Taken together, the most significant risk factors of cancer colon in our study were dietary factors, high BMI, older age, rural residence, cigarette smoking, prolonged intake of NSAIDs and physical inactivity. High serum level of *miRNA-122* is a sensitive and specific for the diagnosis of colon cancer. 

Most of these factors are modifiable which call for a management plan for these risk factors and to decrease the burden of cancer colon. Serum *miRNA-122* with a cut off value 0.946 should be integrated in the primary evaluation of colon cancer patient for early diagnosis & management. Considering the role of *miRNA-122* in promoting the invasiveness and hepatic metastases of colon cancer, the authors recommend further studies & clinical trials to prove the prognostic role of *miRNA-122* in colon cancer.

**Table 1:** Comparison between Cases and Controls Regarding Socio-Demographic Characteristics

Socio-demographic characteristics		Cases (N=102)	Controls (N=102)	Test χ^2^	P
Age (Years)	Mean ±SD	51.47±14.55	39.61±15.11	5.7*	<0.001
Sex	Male	86 (84.3%)	64 (62.7%)	47.38	<0.001
Count (%)	Female	16 (15.7%)	38 (37.3%)		
Residence	Rural	50 (49.0%)	32 (31.4%)	6.6	0.01
Count (%)	Urban	52 (51.0%)	70 (68.6%)		
Marital status	Married	98 (98.0%)	78 (76.5%)	20.87	<0.001
Count (%)	Not married	2 (2.0%)	24 (23.5%)		
Level of education	Read/write and primary	88 (86.3%)	14 (13.7%)	113.123	<0.001
(%)	Preparatory and secondary	8 (7.8%)	14 (13.7%)		
	High	6 (5.9%)	74 (72.6%)		
Employment status	Employed	16 (15.7%)	44 (43.1%)	18.51	<0.001
Count (%)	Unemployed	86 (84.3%)	58 (56.9%)		

*Student t test, P-values less than 0.05 were considered statistically significant

**Table 2:** Lifestyle Habits and Family History in the Studied Groups

Variables			Cases (N=102) Count (%)	Controls (N=102) Count (%)	Test χ^2^	P
Constipation	Never		28 (27.5%)	74 (72.5%)	55.4	<0.001
	Rarely		24 (23.5%)	22 (21.6%)		
	More than once/week		50 (49.0%)	5 (5.9%)		
History of hypercholesterolemia	Yes		46 (45.1%)	26 (25.5%)	8.58	0.003
	No		56 (54.9%)	76 (74.5%)		
Use of NSAIDs	Yes		68 (66.7%)	14 (13.7%)	59.46	<0.001
	No		34 (33.3%)	88 (86.3%)		
Type 2 DM	Yes		82 (80.4%)	40 (39.2%)	35.9	<0.001
	No		20 (19.6%)	62 (60.8%)		
Cigarette smoking	Yes		16 (15.7%)	4 (3.9%)	7.98	0.005
	No		86 (84.3%)	98 (96.1%)		
Physical activity	Physical activity	Vigorous	44 (43.1%)	54 (52.9%)	1.96	0.16
	Count (%)	Moderate and mild	58 (56.9%)	48 (47.1%)		
	Number of days of physical activity as part of work		5 (1-7)*	5 (1-7)*	1.177**	0.239
	Number of hours of physical activity/day as part of work		2 (1-8) *	4 (1-5)*	2.53**	0.01
	Time spent sitting on a typical day	Less than 8 hours	34 (33.3%)	50 (49.0%)	5.18	0.023
	Count (%)	More than 8 hours	68 (66.7%)	52 (51.0%)		
Family history of colon cancer	Yes		22 (21.6%)	24 (23.5%)	0.11	0.738
	No		80 (78.4%)	78 (76.5%)		

*Median and Range, **Mann-Whitney test; N, number

**Table 3:** Dietary Habits and Anthropometric Measurements in the Studied Groups

Variables		Cases (N=102) Count (%)	Controls (N=102) Count (%)	Test χ^2^	P
Red meat intake	< 1 day/ week	64 (62.7%)	92 (90.2%)	21.35	<0.001
	1-3 days/ week	38 (37.3%)	10 (9.8%)		
Processed meat	< 1 day/ week	24 (23.5%)	92 (90.2%)	92.4	<0.001
	1-3 days/ week	78 (76.5%)	10 (9.8%)		
Drinking milk	Never	26 (25.5%)	30 (29.4%)	58.73	<0.001
	Frequent	68 (66.7%)	20 (19.6%)		
	Everyday	8 (7.8%)	52 (51.0%)		
Vegetables and fruits intake	Never	58 (56.9%)	20 (19.6%)	45.39	<0.001
	Frequent	26 (25.5%)	74 (72.5%)		
	Everyday	18 (17.6%)	8 (7.8%)		
Cheese	Never	8 (7.8%)	0 (0.0%)	14.93	<0.001
	Frequent	56 (54.9%)	42 (41.2%)		
	Everyday	38 (37.3%)	60 (58.8%)		
Eggs	Never	18 (17.6%)	8 (7.8%)	59.64	<0.001
	Frequent	76 (74.6%)	34 (33.4%)		
	Everyday	8 (7.8%)	60 (58.8%)		
anthropometric measurements	Weight (kg)	80 (54-102)*	70 (54-96)*	1.84**	0.06
	Height (cm)	163 (150-170)*	165 (150-179)*	3.2**	0.001
	BMI (kg/m^2^)	29.6 (23.5-38.5) *	28 (22.5-35.7)*	2.292**	0.022

*Median and Range, **Mann-Whitney test; N, number, BMI, body mass index

**Table 4:** Comparison between Cases and Controls Regarding *miRNA-122*

Variables	Cases (N=102) Median (Range)	Controls (N=102) Median (Range)	Testχ^2^	P
*miRNA-122*	2.3 (0.63-7.36)	0.39 (0.12-3.19)	11.55*	<0.001

*Mann-Whitney test; miRNA, micro-RNA

**Table 5:** Area under the Receiver Operating Characteristic (AUC ROC) Curve Analysis for *miRNA -122*

	AUC	P	95% confidence interval		Cut off	Sensitivity	Specificity
			Lower	Upper			
*miRNA-122*	0.968	<0.001	0.944	0.993	0.946	95.00%	91.00%

AUC, area under the curve; miRNA, micro-RNA

**Table 6:** Binary Logistic Regression for the Predictors of Cancer Colon

Variables	OR	95% CI	P value
BMI	1.07	1.01 - 1.15	0.031
Fruits and vegetables			
Everyday	1		
Often	1.361	0.349 -5.3	0.657
Never	3.7	1.35 – 10.1	0.01
Age	1.13	1.07 - 1.19	<0.001
Processed meat intake			
1-3 day / week vs < 1 day / week	2.8	1.21 - 6.53	0.016
Residence			
Urban vs rural	2.5	1.03 – 6.1	0.042

## Author Contribution Statement

All authors have substantial contributions in the conception, design, supervision & revision of this manuscript. The manuscript has been read and approved by all authors. MB, and MH were responsible for data collection from patients and statistical analysis. MB, and AM were responsible for diagnosis and recruitment of cases. ME was responsible for blood samples collection and all laboratory investigations.
